# Attention-Based Temporal-Frequency Aggregation for Speaker Verification

**DOI:** 10.3390/s22062147

**Published:** 2022-03-10

**Authors:** Meng Wang, Dazheng Feng, Tingting Su, Mohan Chen

**Affiliations:** National Laboratory of Radar Signal Processing, Xidian University, Xi’an 710071, China; mwang_3@stu.xidian.edu.cn (M.W.); sutingting@stu.xidian.edu.cn (T.S.); mhchen@stu.xidian.edu.cn (M.C.)

**Keywords:** convolutional neural networks, speaker verification, temporal-frequency aggregation, self-attention

## Abstract

Convolutional neural networks (CNNs) have significantly promoted the development of speaker verification (SV) systems because of their powerful deep feature learning capability. In CNN-based SV systems, utterance-level aggregation is an important component, and it compresses the frame-level features generated by the CNN frontend into an utterance-level representation. However, most of the existing aggregation methods aggregate the extracted features across time and cannot capture the speaker-dependent information contained in the frequency domain. To handle this problem, this paper proposes a novel attention-based frequency aggregation method, which focuses on the key frequency bands that provide more information for utterance-level representation. Meanwhile, two more effective temporal-frequency aggregation methods are proposed in combination with the existing temporal aggregation methods. The two proposed methods can capture the speaker-dependent information contained in both the time domain and frequency domain of frame-level features, thus improving the discriminability of speaker embedding. Besides, a powerful CNN-based SV system is developed and evaluated on the TIMIT and Voxceleb datasets. The experimental results indicate that the CNN-based SV system using the temporal-frequency aggregation method achieves a superior equal error rate of 5.96% on Voxceleb compared with the state-of-the-art baseline models.

## 1. Introduction

Speaker verification (SV) is a voice biometric authentication technology developed to judge the claimed identity of a test speaker. With the development of electronic equipment and communication technology, the SV system has been widely used in various fields, such as forensics [1], e-commerce applications [2], general business interactions, and law enforcement [3]. SV can be categorized into text-dependent SV and text-independent SV according to whether the spoken text is restricted or not. This paper focuses on the text-independent SV because it is more challenging and has greater practical significance [4,5]. In real-world conditions, irrelevant signals in speech (e.g., noise, background music) and intraspeaker variability (e.g., emotion, health, age) make it difficult to develop an accurate and robust SV system.

In the past decades, the framework composed of i-vector [6] and probabilistic linear discriminant analysis (PLDA) [7] has dominated the text-independent SV because of its superior performance, simplicity, and efficiency. In this framework, a Gaussian mixture model-universal background model (GMM-UBM) [8] is first used to collect sufficient statistics. Then, a feature extractor (i.e., factor analysis [6]) is used to exact a low-dimensional identity embedding as the compact representation of the utterance. Finally, a separate PLDA classifier is trained to produce verification scores for each pair of utterances. Although the i-vector/PLDA system can achieve great success in some scenarios, the performance of the system decreases when enrollment/test utterance durations are short [9,10].

Recently, several SV systems based on deep neural networks (DNNs) have been developed and have achieved competitive performance compared with traditional i-vector/PLDA systems. Typically, the DNN-based SV method is a neural speaker embedding system. It maps utterances into a feature space, where distances correspond to speaker similarity [11]. To build a neural speaker embedding system, powerful DNN architectures, such as time-delay neural network (TDNN) [12,13], convolutional neural network (CNN) [4,9,14,15,16], and long short-term memory network (LSTM) [17,18], are utilized to extract frame-level features from utterances. Then, the extracted features are compressed into a fixed-length utterance-level representation, and the discriminative speaker embedding is obtained from the bottleneck of the subsequent feed-forward network. In the training process, the DNN-based systems can be trained indirectly via a classification loss, such as softmax loss [15] and angular softmax loss [19]. Some well-designed metric learning losses have been exploited to train the entire SV system in an end-to-end fashion, such as triplet loss [9,11], generalized end-to-end (GE2E) loss [18], and cluster-range loss [4]. Besides, many studies on robust features [20,21] and hybrid models [21,22] have been conducted to further improve the performance of traditional and DNN-based speaker recognition systems. In recent years, CNN has drawn much attention in this research field. Since CNN is excellent in capturing local neighborhood features, compared with the frame-level features in TDNN-based (i.e., x-vector in [12,13]) and LSTM-based SV systems, the two-dimensional (2D) features extracted by CNN can retain the spatial structure and information of time-frequency input (i.e., the order of time frames and frequency bands remains unchanged during the forward propagation). Based on this, not only the speaker-dependent information contained in the time domain but also the information contained in the frequency domain can be exploited to generate more discriminative speaker embeddings.

To better capture speaker characteristics from frame-level features, the utterance-level aggregation has been wildly studied and applied to CNN-based SV systems. Temporal average pooling (TAP) layer [11,19,23] is the most popular aggregation method, which takes the mean of the frame-level features in the time domain as an utterance-level representation. Meanwhile, Snyder et al. [24] employed an extension of TAP, called statistics pooling, in which both the mean and standard deviation are calculated and concatenated to model the speaker characteristics. The standard deviation can reveal any distance in a context, which helps speaker embedding to capture the long-term variability over an utterance [25]. However, the speaker-discriminative power differs between frames of extracted features, so each frame has a different contribution to the utterance-level representation. To address this issue, self-attentive pooling (SAP) [19] and attentive statistics pooling (ASP) [25] have been proposed to adaptively emphasize the discriminative frames. The fundamentals of SAP and ASP are to utilize an attention mechanism to assign different weights to different frames. Besides, Liu et al. [26] presented a unified attention-based pooling framework and combined it with multi-head attention. In addition, some dictionary-based methods have been developed to aggregate features across time, such as learnable dictionary encoding (LDE) [19], net vector of locally aggregated descriptors (NetVLAD) [27], and Ghost VLAD [27]. However, these aggregation methods ignore the speaker-dependent information contained in the frequency domain, limiting the performance of CNN-based SV systems.

In this paper, two temporal-frequency aggregation methods are proposed to overcome the above deficiencies. The methods are composed of two branches: temporal branch and frequency branch. For the frequency branch, a novel shared-parameter grouped frequency self-attentive pooling (SGFSAP) layer is proposed to effectively capture the speaker-dependent information contained in the frequency domain based on the following facts: (1) The speaker-dependent information is distributed in the time domain and frequency domain of the 2D frame-level features generated by the CNN; (2) the individual information is encoded non-uniformly in different frequency bands of utterance [28]; (3) some speaker-dependent frequency information (such as formants information) varies with the phonetic contents of the utterance [26,28,29]. Meanwhile, inspired by the use of self-attention to emphasize the informative frames [19,25], this paper introduces the additive self-attention mechanism [30] to the frequency domain to assign more weights to the frequency bands that provide more information for the utterance-level representation. The frame-level features along the temporal axis are first grouped, and the temporal information within each group is aggregated to obtain a time-varying frequency feature descriptor (FFD) that can sufficiently accumulate the temporal information in each group while adapting to the changes in phonetic contents. Then, self-attention is used to generate attention maps for each group. In addition, considering the occurrence of the same phonetic contents in different groups, the parameters of the self-attention mechanism are shared between groups for further improvement. The shared-parameter method makes SGFSAP invariant to the grouping position (i.e., the position after grouping along the temporal axis). For the temporal branch, any of the previous temporal aggregation methods (such as TAP, SAP, and ASP) can be exploited to model temporal attention. In this work, SGFSAP is combined with SAP and ASP to develop two attention-based temporal-frequency aggregation methods, i.e., SAP-SGFSAP and ASP-SGFSAP, which can capture the speaker-dependent information contained in both the time domain and frequency domain. The main contributions of this paper are summarized as follows.

1. A novel shared-parameter grouped frequency self-attentive pooling layer is proposed to capture the speaker-dependent information contained in the frequency domain.
2. Based on SGFSAP, two temporal-frequency aggregation methods are developed, which can capture the speaker-dependent information contained in both the time and frequency domains, thus obtaining more discriminative utterance-level representation than the original temporal aggregation methods.
3. Based on the modified 34-layer residual network (ResNet-34) [31] architecture (detailed in Table 1) and the proposed temporal-frequency aggregation methods, a powerful CNN-based SV system is constructed for TIMIT [32] and Voxceleb. Meanwhile, the GE2E loss [18] is used for end-to-end optimization of the whole system.
4. Extensive experiments are conducted on the TIMIT and Voxceleb datasets. The experimental results on TIMIT show that the proposed temporal-frequency aggregations are more effective than the temporal aggregations to handle the SV tasks under additive noise and typical distortions. Moreover, the experimental results on Voxceleb indicate that the proposed CNN-based SV system using ASP-SGFSAP achieves an equal error rate (EER) of 5.96% without any data preprocessing technology or complex backend, which is superior to the EER of the state-of-the-art baseline methods. Additionally, compared with the CNN-based SV systems using SAP and ASP, the systems using SAP-SGFSAP and ASP-SGFSAP achieve a relative reduction in EER of 7.14% and 8.87% (lower is better), respectively.
5. The attention maps generated by SAP-SGFSAP and ASP-SGFSAP are visualized and analyzed. The results show that temporal-frequency aggregation methods can capture the frequency information related to the vocal cord and piriform fossa [28], both of which are important for speaker verification. Additionally, these methods can capture some important formants of vowels. Besides, the informative frames are also emphasized in the time domain.

The rest of this paper is organized as follows. Section 2 introduces related works. Section 3 describes the proposed SGFSAP and presents two attention-based temporal-frequency aggregation methods. Section 4 describes the experimental setup and the proposed CNN-based SV systems. The experimental results are visualized and analyzed in Section 5. Finally, Section 6 concludes this paper.

## 2. Related Works

This section introduces the previous studies on the temporal aggregation methods, including attention-based methods and dictionary-based methods, as well as the GE2E loss function used to optimize CNN-based SV systems. First, the notations used in the following descriptions are given. In this study, vectors and matrices are, respectively, represented by lowercase boldface letters (x) and uppercase boldface letters (X) . The superscripts ${\left(\cdot \right)}^{t}$ and ${\left(\cdot \right)}^{f}$ indicate the description of the time domain and the frequency domain, respectively. ${\left(\cdot \right)}^{T}$ represents the transpose. Suppose that the set of frame-level features generated by the CNN frontend are $\left\{x_{f,t}\right\}$ ( $f\in \left[1,F\right]$ , $t\in \left[1,T\right]$ ), where *F* and *T* are the dimensions of the frequency and temporal axes, respectively. $x_{f,t}\in R^{C\times 1}$ is the frame-level feature located in the f -th frequency band and t -th frame, *C* denotes the number of channels. For simplicity, the frame-level features are formulated as a matrix $X\in R^{C\times F\times T}$ . Typically, an average pooling layer [19] or a fully connected layer [15,27] is used along the frequency axis of the extracted 2D features to generate a temporal feature descriptor (TFD) matrix $X^{t}\in R^{C\times T}$ , and $X^{t}=\left[x_{1}^{t},x_{2}^{t},\ldots ,x_{T}^{t}\right]$ , where $x_{t}^{t}\in R^{C\times 1}$ . In this paper, the average pooling is used to generate a TFD matrix for temporal aggregation because it is simple and efficient.

### 2.1. Attention-Based Temporal Aggregation

#### 2.1.1. Self-Attentive Pooling

By introducing self-attention into the time domain, Cai et al. [19] proposed an SAP layer to pay attention to the frames that are important to speaker recognition and use the weighted mean of frames to form an utterance-level representation. The calculation of temporal attention map $\alpha^{t}\in R^{T\times 1}$ and utterance-level representation $e\in R^{C\times 1}$ is shown as follows:

(1) $$h_{t}=\tanh\left({\mathrm{Wx}}_{t}^{t}+b\right)$$

(2) $$\alpha_{t}^{t}=\frac{\exp({h_{t}}^{T}v)}{\sum_{t=1}^{T}\exp({h_{t}}^{T}v)}$$

(3) $$e=\sum_{t=1}^{T}\alpha_{t}^{t}x_{t}^{t}$$

where $W\in R^{C\times C}$ , $b\in R^{C\times 1}$ , $v\in R^{C\times 1}$ are trainable parameters. $\alpha_{t}^{t}$ is the attention score for the t -th frame of the frame-level features. As discussed in [19], the vector v can be considered a fixed query, “What is the informative frame over the whole time domain?”.

#### 2.1.2. Attentive Statistics Pooling

The implementation of the ASP layer [25] uses the same self-attention mechanism as SAP to calculate the attention score (also called weight in some literature) for each frame. Unlike SAP, in ASP, the weighted standard deviation and the weighted mean of frame-level features are calculated and concatenated to generate the utterance-level representation $e\in R^{2C\times 1}$ . The calculation process is shown as follows:

(4) $$\tilde{\mu }=\sum_{t=1}^{T}\alpha_{t}^{t}x_{t}^{t}$$

(5) $$\tilde{\sigma }=\sqrt{\sum_{t=1}^{T}\alpha_{t}x_{t}⊙x_{t}-\tilde{\mu }⊙\tilde{\mu }}$$

(6) $$e=[\tilde{\mu },\tilde{\sigma }]$$

where the temporal attention score $\alpha_{t}^{t}$ is calculated by Equations (1) and (2), and ⊙ represents the Hadamard product. It should be noted that the attention scores are shared between the weighted mean and the weighted standard deviation.

### 2.2. Dictionary-Based Temporal Aggregation

#### 2.2.1. NetVLAD

NetVLAD [33] is an effective method to obtain image representation in computer vision. Xie et al. [27] introduced NetVLAD into speaker verification and achieved good performance. Given a TFD matrix $X^{t}$ as input and *K* cluster centers $\left\{c_{k}\right\}$ as VLAD parameters, the output of NetVLAD can be expressed as a matrix $U\;=\;[u_{1}^{T},u_{2}^{T},\ldots ,u_{K}^{T}{]}^{T}$ with a size of K×C , and the element at the position of (k,j) is obtained by the following equation:

(7) $$U\left(k,j\right)=\sum_{t=1}^{T}\frac{e^{w_{k}^{T}x_{t}^{t}+b_{k}}}{\sum_{k^{\prime }=1}^{K}e^{w_{k^{\prime }}^{T}x_{t}^{t}+b_{k}}}\left[x_{t}^{t}\left(j\right)-c_{k}\left(j\right)\right]$$

where $w_{k}$ , $b_{k}$ and $c_{k}$ are trainable parameters, with k∈[1,2,…,K] . The first term corresponds to the soft-assignment weight of the input feature vector $x_{t}^{t}$ in cluster *k*, and the second term represents the residual between the feature vector and the cluster center [27,34]. Finally, the matrix U is converted into a vector, and L2-normalization is conducted to obtain the final utterance-level representation $e\in R^{KC\times 1}$ as follows:

(8) $$e=\frac{\left[u_{1},u_{2},\ldots ,u_{K}\right]}{{∥\left[u_{1},u_{2},\ldots ,u_{K}\right]∥}_{2}}$$

#### 2.2.2. GhostVLAD

GhostVLAD [27] is similar to NetVLAD, except that the number of chosen clusters is K+G . Additionally, in GhostVLAD, the residuals between input feature vectors and *G* “ghost” cluster centers are discarded and do not contribute to the utterance-level representation (see more details in [27,34]).

### 2.3. Generalized End-to-End Loss

In this work, the GE2E loss function [18,35] is adopted to train all CNN-based SV systems. For each training step, a mini-batch contains *M* utterances from *N* different speakers. The speaker embeddings generated by CNN-based SV systems are denoted as $e_{ji}$ , where 1⩽j⩽N and 1⩽i⩽M . The centroid of the *M* utterances from speaker *j* is defined as

(9) $$c_{j}=\frac{1}{M}\sum_{m=1}^{M}e_{jm}$$

The similarity matrix $\left\{S_{ji,k}\right\}$ is defined as scaled cosine similarity between each speaker embedding vector $e_{ji}$ and all centroids $c_{k}$ , with 1⩽j,k⩽N and 1⩽i⩽M . Meanwhile, the embedding $e_{ji}$ is removed when calculating the centroid of the true speaker. Hence, the following equations can be obtained:

(10) $$c_{j}^{\left(-i\right)}=\frac{1}{M-1}\sum_{m=1,m\neq i}^{M}e_{jm}$$

(11) $$S_{ji,k}=\left\{\begin{array}{c} w\cdot \cos(e_{ji},c_{j}^{\left(-i\right)})+b\;\mathrm{if}\;k=j;\\ w\cdot \cos(e_{ji},c_{k})+b\;\mathrm{otherwise}. \end{array}\right.$$

where w>0 and *b* are trainable parameters. The final GE2E loss is defined as the sum of all losses over the similarity matrix:

(12) $$L=\sum_{j,i}L(e_{ji})=\sum_{j,i}\left[-S_{ji,j}+\log\sum_{k=1}^{N}\exp(S_{ji,k})\right]$$

## 3. Proposed Methods

In this section, the SGFSAP method is first proposed. Then, two temporal-frequency aggregation methods, i.e., SAP-SGFSAP and ASP-SGFSAP, are proposed to capture the speaker-dependent information contained in both the time domain and frequency domain. Furthermore, a powerful CNN-based speaker verification scheme is developed for the TIMIT and Voxceleb datasets.

### 3.1. SGFSAP

In CNN-based SV systems, it is difficult for existing aggregation methods to effectively capture the speaker-dependent information contained in the frequency domain of frame-level features. To handle this problem, this paper introduces a self-attention mechanism in the frequency domain and proposes a novel attention-based frequency aggregation method.

**Figure 1:**
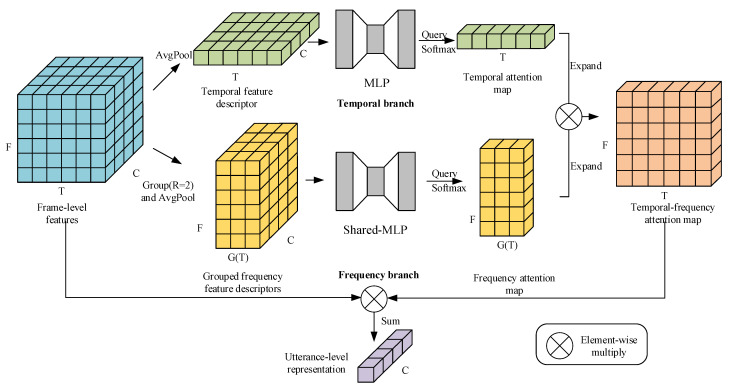
Diagram of the attention-based temporal-frequency aggregation method.

Suppose that the set of frame-level features extracted by CNN frontend are $\left\{x_{f,t}\right\}$ ( $f\in \left[1,F\right]$ and $t\in \left[1,T\right]$ ), which can be formulated as a matrix $X\in R^{C\times F\times T}$ (see more details in Section 2). To model frequency attention, the receptive field of the attention module needs to focus only on the frequency axis of frame-level features [36]. An intuitive method is to aggregate the temporal information through global average pooling, and this method can generate a frequency feature descriptor (FFD) matrix $X^{f}\in R^{C\times F}$ . However, as mentioned in Section 1, some speaker-dependent frequency information (such as formants information) varies with the phonetic contents of the utterance, i.e., the information is almost constant in the short time but varies over a long time. To address these issues, this paper divides the frame-level features into *G* groups along the temporal axis and then aggregates the temporal information within each group using an average pooling layer, as illustrated in Figure 1. Here, G=TTRR indicates the number of groups, where $\left\lceil \cdot \right\rceil$ represents the ceiling operation, and *R* denotes the number of frames aggregated in each group (also called grouped-ratio), and it is a predefined hyperparameter. After this, an effective FFD matrix $X^{f}\in R^{C\times F\times G}$ and $X^{f}=\left[X_{1}^{f},X_{2}^{f},\ldots ,X_{G}^{f}\right]$ can be obtained, which is composed of *G* grouped-FFDs (GFFDs). $X_{g}^{f}\;=\;\left[x_{1,g}^{f},x_{2,g}^{f},\ldots ,x_{F,g}^{f}\right]$ ( $g\in \left[1,G\right]$ ) denotes the g -th GFFD.

For an utterance, it is assumed that the same phonetic contents in different groups lead to similar spectral structure changes in the frequency domain. This requires the frequency aggregation method to be group-invariant. To this end, this paper shares the parameters of the attention mechanism between groups. Specifically, additive self-attention [30] is introduced into the frequency domain of each group. As illustrated in Figure 1, a shared multi-layer perceptron (shared-MLP) is adopted to calculate the compatibility function map for each group, i.e., the parameters of MLP are shared between different groups. The shared-parameter method enables SGFSAP to have group-invariance so that it can effectively capture the speaker-dependent frequency information generated by the same phonetic contents in different groups. According to the description above, the frequency attention map of the g -th group $\alpha_{g}^{f}\in R^{F\times 1}$ ( $g\in \left[1,G\right]$ ) can be calculated as follows:

(13) $$h_{f,g}^{f}=\tanh\left({\mathrm{Wx}}_{f,g}^{f}+b\right)$$

(14) $$\alpha_{f,g}^{f}=\frac{\exp({\left(h_{f,g}^{f}\right)}^{T}v^{f})}{\sum_{f=1}^{F}\exp({\left(h_{f,g}^{f}\right)}^{T}v^{f})}$$

where $W\in R^{C\times C}$ and $b\in R^{C\times 1}$ denote the parameters of MLP, and they are shared between groups; $\alpha_{f,g}^{f}$ is the frequency attention score of the g -th group in the f -th frequency band; $v^{f}\in R^{C\times 1}$ represents a query vector, and it is randomly initialized and jointly learned during the training process. It should be noted that $v^{f}$ is group-independent.

It can be seen that the hidden representation $h_{f,g}^{f}\in R^{C\times 1}$ summarizes the frequency information of the f -th band in the g -th GFFD. In this paper, the frequency attention score $\alpha_{f,g}^{f}$ is generated by measuring the importance of the f -th frequency band as the similarity between $h_{f,g}^{f}$ and a query vector $v^{f}$ . Inspired by the description of the query vector in temporal aggregation [19], the vector $v^{f}$ can be considered a high-level representation of a fixed query “What is the informative frequency band over the whole frequency domain in each group?”.

Given the frequency attention map of each group, the frequency attention map of frame-level features can be formulated as $A^{f}\in R^{F\times G}$ and $A^{f}=\left[\alpha_{1}^{f},\alpha_{2}^{f}\cdots \alpha_{G}^{f}\right]$ . Since $A^{f}$ and X have different shapes, the frequency attention map is expanded to a F×T matrix using the same padding along the temporal axis within each group. It is implicitly assumed that the speaker-dependent frequency information is almost constant within each group and varies from group to group. Hence, the utterance-level representation $e\in R^{C\times 1}$ can be generated as a weighted mean of frame-level features:

(15) $$e=\sum_{t=1}^{T}\sum_{f=1}^{F}\alpha_{f,t}^{f}x_{f,t}$$

Here, $x_{f,t}$ indicates the feature vector of the f -th frequency band and the t -th time frame of the frame-level features, and its frequency attention score is $\alpha_{f,t}^{f}$ .

### 3.2. SAP-SGFSAP and ASP-SGFSAP

**Figure 2:**
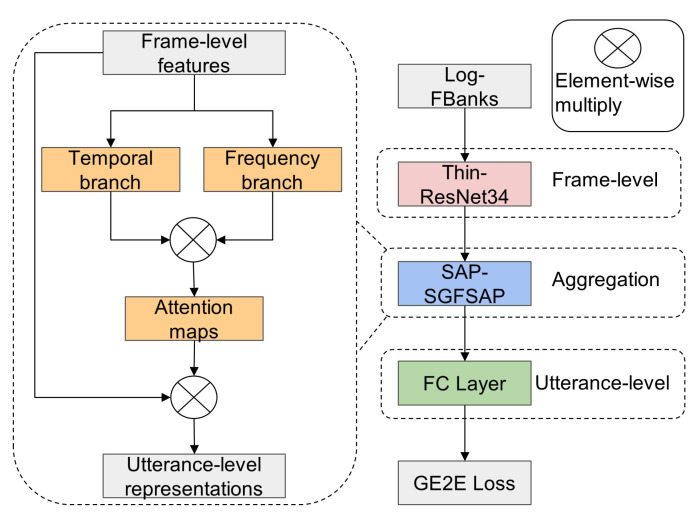
The CNN-based SV system using SAP-SGFSAP.

In this subsection, two attention-based temporal-frequency aggregation methods are developed: SAP-SGFSAP and ASP-SGFSAP. Specifically, SGFSAP is used to build the frequency branch, and SAP or ASP is used to build the temporal branch, as shown in Figure 1 and Figure 2. Attributed to their parallel structure, the two temporal-frequency aggregation methods can simultaneously capture the speaker-dependent information contained in the time domain and frequency domain, thus obtaining more discriminative utterance-level representations than temporal aggregations. Note that SGFSAP can be used alone and achieves better performance than all temporal aggregation methods, which will be shown in the following.

The key of SAP-SGFSAP and ASP-SGFSAP is to generate a temporal-frequency attention map for frame-level features. Given a temporal attention map $\alpha^{t}$ generated by SAP or ASP (detailed in Section 2.1) and a frequency attention map $A^{f}$ generated by SGFSAP, this paper first expands the two attention maps to $R^{F\times T}$ because of the different shapes of them. As described in Section 3.1, $A^{f}=\left[\alpha_{1}^{f},\alpha_{2}^{f}\cdots \alpha_{G}^{f}\right]$ , and $\alpha_{g}^{f}$ ( $g\in \left[1,G\right]$ ) is expanded into an F×R matrix using the same padding along the temporal axis. Here, *R* is the number of frames in each group. Thus, a frequency attention map with a size of F×T can be obtained. Then, $\alpha^{t}$ is expanded into an F×T matrix using the same padding along the entire frequency axis. Finally, the expanded attention maps are combined with element-wise multiplication to generate the final temporal-frequency attention map $A\in R^{F\times T}$ , which is channel-independent and group-varying.

Based on the temporal-frequency attention map A , the weighted mean $\tilde{\mu }$ and the weighted standard deviation $\tilde{\sigma }$ of frame-level features are defined as

(16) $$\tilde{\mu }=\sum_{t=1}^{T}\sum_{f=1}^{F}\alpha_{f,t}x_{f,t}$$

(17) $$\tilde{\sigma }=\sqrt{\sum_{t=1}^{T}\sum_{f=1}^{F}\alpha_{f,t}x_{f,t}⊙x_{f,t}-\tilde{\mu }⊙\tilde{\mu }}$$

where $\alpha_{f,t}$ is the temporal-frequency attention score of $x_{f,t}$ .

In SAP-SGFSAP, the weighted mean vector focusing on the important frequency bands and time frames is used as the utterance-level representation $e\in R^{C\times 1}$ , which can be formulated as

(18) $$e=\tilde{\mu }$$

Meanwhile, in ASP-SGFSAP, the weighted mean and the weighted standard deviation are contacted and used as the utterance-level representation $e\in R^{2C\times 1}$ , which can utilize the higher-order statistics [25] in the frame-level features.

(19) $$e=\left[\tilde{\mu },\tilde{\sigma }\right]$$

### 3.3. CNN-Based Speaker Verification Systems

In this subsection, an end-to-end CNN-based speaker embedding scheme is proposed for speaker verification on TIMIT and Voxceleb. As illustrated in Figure 2, the system consists of three parts: frame-level part, aggregation part, and utterance-level part.

- **Frame-level part** In this work, Thin ResNet-34 (detailed in Table 1) is adopted as the trunk architecture to extract the frame-level features because of its strong ability in learning deep features. Thin ResNet-34 is the same as the original 34-layer ResNet [31] except that it uses only one-quarter of the channels in each residual block to reduce the number of parameters and computational cost [35]. The standard ResNet-34 has 22 million parameters, while the Thin ResNet-34 has only about 1.35 million parameters.
- **Aggregation part** The proposed SAP-SGFSAP and ASP-SGFSAP are used to aggregate the frame-level features. For simplicity, the values of *R* that can divide *T* are used in this work, i.e., R=1,2,4,19,38,76 for T=76 . Meanwhile, temporal aggregation methods are adopted to aggregate frame-level features across time. Specifically, an average pooling layer is exploited to produce an utterance-level feature map with a size of 128×1×TT44 . Then, the temporal aggregation methods are used for aggregation, including TAP, SAP, ASP, NetVLAD ( K=8 ), and GhostVLAD ( K=8,G=2 ).
- **Utterance-level part** The utterance-level part consists of a fully connected (FC) layer with D-dimensional output, which transforms the utterance-level representation into a compact speaker embedding. In this paper, 256-dimensional speaker embedding (i.e., D=256 ) is used.

Subsequently, the simple cosine similarity between speaker embeddings is used to generate a score for each pair of utterances. Finally, the GE2E loss [18,35] is used to optimize the entire system in an end-to-end manner.

## 4. Experimental Setup

### 4.1. Dataset and Input Feature

In this paper, speaker verification experiments are conducted on TIMIT [32] and Voxceleb [15] datasets. TIMIT is a clean speech dataset that contains 10 sentences each spoken by 630 speakers, a total of 6300 utterances. For the SV task, 462 speakers are selected for training, and the other 168 speakers are selected for testing. Voxceleb is a large-scale text-independent speaker verification dataset containing over 100,000 utterances from 1251 speakers. Unlike TIMIT, Voxceleb was collected under multimedia acoustic conditions. Particularly, all the audios in Voxceleb are mixed with real-world noise, including background chatter, laughter, overlapping speech, and room acoustics [15]. In addition, the quality of the recording equipment and channel noise differs. Following the data splitting scheme in [15], 1211 speakers are selected for training, and the other 40 speakers whose names start with an “E” are selected for testing. There is no overlap between the testing and training sets.

The proposed CNN-based SV systems take log Fbank coefficients as their input. In the experiments on Voxceleb, to generate a time-frequency input, audio of 3 s is randomly clipped from an utterance, and it is first framed with a hamming window (25 ms duration and 10 ms shift) to obtain 300 frames of data. Then, each frame is converted into 64-dimensional Fbank coefficients, and a log operation is performed to obtain the final log FBank coefficients with a size of 64×300 . In the experiments on TIMIT, the input features are obtained in the same way as those on Voxceleb, except that the length of input is set to 2 s. It should be noted that no data preprocessing techniques (such as voice activity detection and data augmentation [12]) are employed in our experiments except random clipping.

### 4.2. Baselines

To comprehensively evaluate the proposed aggregation methods and the CNN-based SV systems, the following baselines are taken for performance comparison:

- **GMM-UBM** The GMM-UBM system uses mel frequency cepstrum coefficients with a dimension of 13 as input. Cepstral mean and variance normalization is applied to the features. The UBM with 1024 mixture components is trained for 10 iterations with the training data [15].
- **I-vector/PLDA** Gender-independent i-vector extractors are trained to produce 400-dimensional i-vectors. Then, PLDA is used to reduce the dimension of the i-vectors to 200, see more details in [15].
- **CNN-embedding** In [15], a modified VGG-M is used to extract compact speaker embeddings. The network takes spectrograms with a size of 512×300 as input, which are extracted from randomly clipped 3-second audios. The entire system is optimized using a contrastive loss.
- **X-vector** X-vector is the state-of-the-art DNN-based model for speaker verification [4]. This paper collects the results of the x-vector systems using cosine and PLDA backends from the reference [37].
- **CNN-based SV systems** The CNN-based SV systems using temporal aggregation methods (such as SAP, ASP, NetVLAD, and GhostVLAD) are used as baselines to verify the effectiveness of the proposed aggregation methods; see more details in Section 3.3.

### 4.3. Details

In this study, an RMSprop [38] optimizer is employed to optimize each CNN-based SV system. In the experiments on Voxceleb, each mini-batch contains 10 speakers ( N=10 ), and each speaker has 10 segments ( M=10 ) during training, i.e., 100 segments for each batch. The learning rate is initialized to 0.0001, and it decreases by a factor of 10 every 500 epochs until 2000 epochs. The experimental results indicate that the systems converge stably after 1000 epochs, so the training time can be reduced by stopping the training phase early. In the experiments on TIMIT, each speaker has six segments in the training phase, i.e., 60 segments for each batch. The learning rate decreases by a factor of 10 every 100 epochs until 500 epochs. In the test set, the number of enrolling utterances for each speaker is 1. All the CNN-based SV systems are implemented in PyTorch [39] and run on two 1080Ti GPUs.

For CNN-based SV systems, the evaluation protocol proposed in [16] is adopted to evaluate their performance at test time. In the experiments on Voxceleb, 10 temporal crops of 3 s are sampled at a regular interval in each test utterance. Then, the cosine distances between every possible pairwise crop ( 10×10=100 ) are calculated, and the mean of the 100 distances is taken as the score. Similarly, the same evaluation protocol is used in the experiments on TIMIT, except that the length of crops is 2 s. Finally, the EER is reported to verify the performance of these systems on the TIMIT and Voxceleb datasets.

## 5. Experimental Results

### 5.1. Experiments under Different SNR Levels and Typical Distortions

To verify the effectiveness of the proposed temporal-frequency aggregations and CNN-based SV systems in the noisy condition, extensive experiments are conducted on TIMIT with different signal-to-noise ratio (SNR) levels, i.e., SNR = 10 dB, 20 dB, 30 dB, obtained by adding white Gaussian noise to each audio. Meanwhile, the proposed methods are compared with the basic approaches for typical distortions of speech acquisition, e.g., variation of microphone–mouth distance and room reverberation. Acoustic simulations of reverberant environments were obtained with the image-source method [40] in a room of 4.5 m × 3.75 m × 3.05 m. An omnidirectional microphone is placed at (0.8 m, 1.875 m, 1.2 m), and the sound source (speaker) is located at a random coordinate (x,y,z) to simulate the variation of microphone-mouth distance. Here, $x\in U\left(0.5,4.0\right)$ , $y\in U\left(0.5,3.25\right)$ and $z\in N\left(1.75,0.1\right)$ , where $U\left(\cdot \right)$ and $N\left(\cdot \right)$ represent uniform distribution and Gaussian distribution respectively. The experimental results (EER (%)) are reported in Table 2.

Hereinafter, the proposed CNN-based SV systems (i.e., the CNN-based SV system using different aggregation methods) are represented by their aggregation methods. Meanwhile, the numbers after SGFSAP, SAP-SGFSAP, and ASP-SGFSAP indicate the value of *R* used in the experiments. For example, ASP-SGFSAP-2 indicates the CNN-based SV system uses ASP-SGFSAP with R=2 . In all experiments, the performance using quasi-optimal *R* is reported unless otherwise stated. The NetVLAD-based and GhostVLAD-based approaches are not compared here due to their poor performance on long time frames (50 frames, only 8 frames in reference [27]; see more details in Section 5.3).

By comparing columns 2 to 5 in Table 2, the performance of the CNN-based SV systems decreases as the SNR varies from 30 dB to 10 dB. The SV systems using the proposed temporal-frequency aggregations significantly outperform the counterpart systems using basic aggregation methods under different SNR levels, especially in the case of low SNR level (10 dB). Meanwhile, compared to the clean speech condition, the EER of CNN-based SV systems increases significantly (lower is better) in typical distortion conditions, and the SAP-SGFSAP-10 system achieves the best EER of 7.48% in typical distortion conditions. In addition, ASP-SGFSAP-2 achieves poor performance under typical distortions. We argue that this is because the data volume is too small to capture the statistical information about speakers. The experimental results show that the proposed temporal-frequency aggregation methods are more effective than the basic aggregation methods to handle the SV tasks under additive noise and typical distortions.

### 5.2. Experiments for Various Speaker Verification Systems

To verify the effectiveness of the proposed CNN-based SV systems, they are compared with the following four SV systems: GMM-UBM [15], i-vector/PLDA [15], x-vector (cosine) [37], and x-vector (PLDA) [37]. The cosine and PLDA in parentheses denote the backend modules.

It can be seen from Table 3 that all the proposed CNN-based systems are significantly superior to GMM-based systems and TDNN-based systems. Particularly, the ASP-SGFSAP-1 system achieves the best EER of 5.96%, 16.1% less than that of the state-of-the-art x-vector (PLDA) system. This demonstrates the effectiveness of the proposed CNN-based SV systems. Additionally, it can be seen that, compared with the modified VGG-M [15]. Thin ResNet-34 can generate more powerful frame-level features for utterances, thus improving the performance of speaker verification. In addition, it is observed that the performance improvement of the x-vector (PLDA) over the x-vector (cosine) relies on the complex PLDA backend [7]. In contrast, the proposed CNN-based SV systems simply use the cosine similarity between the pair of speaker embeddings, but this achieves surprising results. This demonstrates that the proposed CNN-based SV systems including SGFSAP-19, SAP-SGFSAP-19, and ASP-SGFSAP-1 can generate more discriminative speaker embeddings than the x-vector (PLDA) system, and they do not require a complex backend.

The CNN-based residual self-attention (Res-SA) [4] model obtains state-of-the-art performance on Voxceleb. An EER of 6.1% (the EER of ASP-SGFSAP-1 is 5.96%) is obtained when a batch hard triplet loss [41] is used in the training phase. Meanwhile, a more efficient cluster-range loss [4] is used to directly decrease the intraclass variation and increase the interclass distance between speaker embeddings and further improve the performance of the Res-SA model, achieving the best EER of 5.5%. In actuality, the performance of the proposed CNN-based SV systems can also be improved by training with more efficient loss functions and some other tricks in the training phase; however, this is beyond the scope of this paper and will be studied in the future.

### 5.3. Experiments for Various Aggregation Methods

To validate the effectiveness of the proposed temporal-frequency aggregation methods, extensive experiments are conducted for the CNN-based SV systems using different aggregation methods on Voxceleb. The experimental results are reported in Table 4. From Table 4, the following observations can be made:

1. The SGFSAP-19 system performs better in EER than all temporal aggregation baselines. This indicates that the speaker-dependent information contained in the frequency domain is crucial for the generation of utterance-level representations. Additionally, our proposed SGFSAP can effectively compress the speaker-dependent frequency information into a compact utterance-level representation.
2. By combining SGFSAP with SAP and ASP, SAP-SGFSAP and ASP-SGFSAP further improve the performance of CNN-based SV systems. Specifically, compared with SGFSAP-19, SAP-SGFSAP-19 and ASP-SGFSAP-1 reduce the EER by 2.40% and 4.79%, respectively. Hence, the temporal branch is important in temporal-frequency aggregation methods.
3. Compared with SAP and ASP, the systems SAP-SGFSAP-19 and ASP-SGFSAP-1 reduce the EER by 7.14% and 8.87%, respectively. The results demonstrate that SGFSAP is an effective and important branch (frequency branch) in temporal-frequency aggregation methods. Additionally, SGFSAP can boost the performance of attention-based temporal aggregation methods.
4. Compared with SAP-SGFSAP-19, the ASP-SGFSAP-1 system uses both weighted mean and weighted standard deviation as utterance-level representation, and it achieves better performance in terms of EER (6.11% vs. 5.96%). This suggests that the standard deviation contains other speaker characteristics in terms of temporal variability over long contexts [25]. These long-term speaker characteristics are important for the speaker verification task.

Furthermore, it can be observed that the systems using NetVLAD and GhostVLAD yield slightly poor results. This may be because the frame-level features generated by Thin ResNet-34 have too many frames (64 frames, only 8 frames in reference [27]). In this case, it is difficult to assign feature vectors to the selected cluster, resulting in performance degradation.

**Figure 3:**
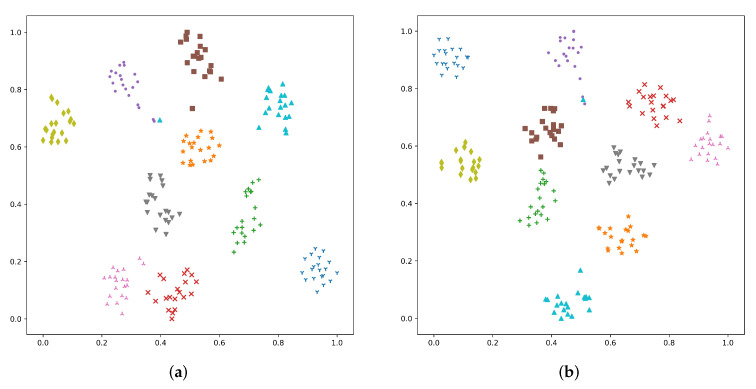

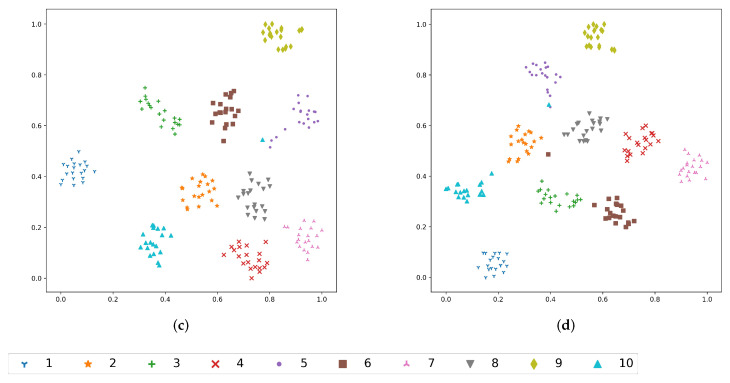
Two-dimensional representation of the speaker embeddings generated by various CNN-based SV systems: (**a**) SAP, (**b**) SAP-SGFSAP-19, (**c**) ASP, (**d**) ASP-SGFSAP-1.

To further investigate temporal-frequency aggregation methods, the speaker embeddings generated by four CNN-based SV systems are visualized, including SAP, SAP-SGFSAP-19, ASP, and ASP-SGFSAP-1. Figure 3 shows the visualization results of the 10 speakers selected from the Voxceleb test set, and each speaker has 20 utterances. All speaker embeddings are projected into a 2D subspace by the t-SNE [42] algorithm, and different speakers are marked with different colors. Figure 3 shows that although SAP and ASP can generate discriminative speaker embeddings, SAP-SGFSAP-19 and ASP-SGFSAP further gather the embeddings of the same speakers (such as speaker 3, 6, 8 in SAP and SAP-SGFSAP-19, speaker 4, 5, 7 in ASP and ASP-SGFSAP-1). This indicates that SGFSAP can capture the speaker-dependent information contained in the frequency domain to improve the discriminability of speaker embeddings generated by SAP and ASP.

### 5.4. Ablation Studies

In this section, two sets of experiments are conducted to show the effectiveness of our design choices in SGFSAP.

#### 5.4.1. Effectiveness of Shared-Parameter

To verify the contribution of shared-parameter in frequency aggregation, a grouped frequency self-attentive pooling (GFSAP) layer is built, where the parameters of MLPs between groups are not shared, i.e., different MLPs are used for different groups of GFFDs ( $X_{g}^{f}$ , with $g\in \left[1,G\right]$ ) to calculate the hidden representations. Thus, the frequency attention map $\alpha^{f}$ is defined as follows:

(20) $$h_{f,g}^{f}=\tanh\left(W_{g}x_{f,g}^{f}+b_{g}\right)$$

(21) $$\alpha_{f,g}^{f}=\frac{\exp({\left(h_{f,g}^{f}\right)}^{T}v_{g}^{f})}{\sum_{f=1}^{F}\exp({\left(h_{f,g}^{f}\right)}^{T}v_{g}^{f})}$$

where $W_{g}\in R^{C\times C}$ and $b_{g}\in R^{C\times 1}$ denote the trainable parameters of MLP in the g -th group, and $v_{g}^{f}$ is a group-dependent query vector. Analogously, $v_{g}^{f}$ can be considered a high-level representation of a fixed query “What is the informative frequency band over the whole frequency domain in the g -th group?”.

Similarly, by combining GFSAP with SAP, a temporal-frequency aggregation method called SAP-GFSAP can be obtained. In ablation experiments, R=19 is set for all the aggregation methods. Table 5 presents the experimental results on Voxceleb of four SV systems: GFSAP-19, SGFSAP-19, SAP-GFSAP-19, and SAP-SGFSAP-19.

As shown in Table 5, the shared-parameter method can reduce the EER of GFSAP-19 from 6.40% to 6.26% and that of SAP-GFSAP-19 from 6.30% to 6.11%. The results indicate that the shared-parameter method is effective and important for the proposed aggregation methods, which enables SGFSAP and SAP-SGFSAP to capture the speaker-dependent frequency information generated by the same phonetic contents in different groups.

Furthermore, the number of parameters of SGFSAP and GFSAP can be calculated as follows:

(22) $$\#\;Paramas\left(\mathrm{SGFSAP}\right)=C^{2}+2C$$

(23) $$\#\;Params\left(\mathrm{GFSAP}\right)=G\times \left(C^{2}+2C\right)$$

where *C* denotes the number of channels. When *C* is given, #Params(GFSAP) is proportional to the number of groups, while #Params(SGFSAP) is a constant. Hence, the SGFSAP and SGFSAP-based temporal-frequency aggregation methods can be implemented at a negligible overhead.

#### 5.4.2. Effectiveness of Grouping Method

**Figure 4:**
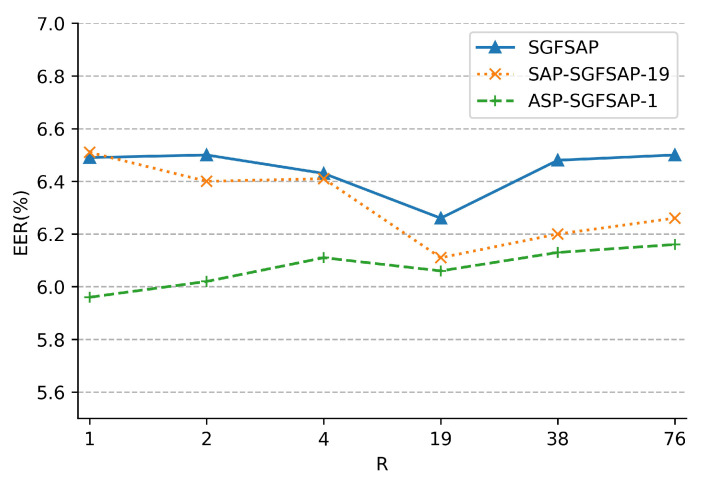
The effectiveness of grouping method (EER versus *R*).

In this subsection, the CNN-based systems using SGFSAP, SAP-SGFSAP, and ASP-SGFSAP with different values of *R* (i.e., R=1,2,4,19,38,76 , T=76 ) are compared to validate the effectiveness of the grouping method. When R=76 , SGFSAP, SAP-SGFSAP, and ASP-SGFSAP become the aggregation methods without grouping. Figure 4 shows the variation of EER with *R* of different SV systems. From Figure 4, the following observations can be made:

1. The best performance of CNN-based systems using SGFSAP, SAP-SGFSAP, and ASP-SGFSAP is achieved at R=19 , R=19 , and R=1 , respectively. The results indicate that finding the optimal value of *R* is essential to obtaining an excellent SV system. This also provides a set of quasi-optimal values of *R* for the CNN-based SV systems on Voxceleb. Besides, the systems of SGFSAP-19, SAP-SGFSAP-19, and ASP-SGFSAP-1 perform significantly better than their counterparts without grouping (i.e., SGFSAP-76, SAP-SGFSAP-76, and ASP-SGFSAP-76). Therefore, grouping is important for the proposed aggregation methods.
2. The EER of the systems using SGFSAP and SAP-SGFSAP decreases firstly and then increases when the value of *R* increases from 1 to 76. For the Voxceleb dataset, it is argued that a small value of *R* (such as R⩽4 ) is favorable for SGFSAP and SAP-SGFSAP to capture the group-varying frequency information in the utterance. However, enough temporal information cannot be accumulated in each group to generate an effective frequency feature descriptor. On the contrary, when the value of *R* is large (such as R⩾38 ), SGFSAP and SAP-SGFSAP can accumulate sufficient temporal information in each group. However, the speaker-dependent frequency information that changes with the phonetic contents is discarded. Especially, SGFSAP-19 and SAP-SGFSAP-19 can fully accumulate the temporal information in each group while adapting to the changes of phonetic contents.
3. Different from SGFSAP and SAP-SGFSAP, the best performance of ASP-SGFSAP is obtained at R=1 . As discussed in [25], it is believed that the standard deviation contains other speaker characteristics in terms of temporal variability over long contexts and plays an important role in utterance-level representation. Thus, the standard deviation can provide long-term temporal information for ASP-SGFSAP. Besides, a small value of *R* helps ASP-SGFSAP to capture the speaker-dependent information that changes with the phonetic contents. As a result, ASP-SGFSAP can achieve better performance when the value of *R* is small. Additionally, it can be observed that ASP-SGFSAP is more robust to the value of *R* than SGFSAP and SAP-SGFSAP. Therefore, for a new dataset, it is recommended to use ASP-SGFSAP instead of SAP-SGFSAP in a CNN-based SV system. Meanwhile, searching for the optimal *R* can start from a small value to speed up the search process.

### 5.5. Visualization and Analyses

**Figure 5:**
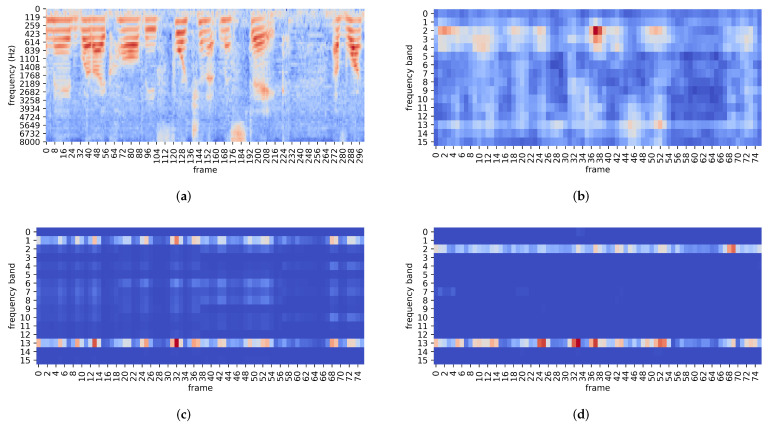
Visualization of the intermediate results of CNN-based SV systems: (**a**) log Fbank coefficients, (**b**) the mean of frame-level features in the ASP-SGFSAP-1 system, (**c**) the attention map generated by SAP-SGFSAP-19, (**d**) the attention map generated by ASP-SGFSAP-1.

To validate the effectiveness of temporal-frequency aggregation methods in capturing the speaker-dependent information contained in both frequency and time domains, the attention maps generated by SAP-SGFSAP-19 and ASP-SGFSAP-1 are visualized in Figure 5. Additionally, the log Fbank coefficients and the mean of frame-level features are visualized to explore the information that can be captured by temporal-frequency aggregation methods. In this figure, a warmer color represents a larger value. The audio segment of 3 s used in the visualization is randomly cropped from an utterance in Voxceleb. The tokens (i.e., phonetic contents) of this segment are “made what I call the fortunate mistake of watching”. According to Table 1 and Section 3.3, the attention maps generated by SAP-SGFSAP-19 and ASP-SGFSAP-1 for the segment have a size of 16×76 , representing 16 frequency bands and 76 time frames.

In Figure 5a, a large number of white scattered spots can be observed. This suggests that there is a lot of noise in the input audio, which is actually a female voice with musical background noise. As can be seen from Figure 5a,b, the Thin ResNet-34 frontend of the ASP-SGFSAP-1 system can capture some important temporal-frequency information from the input, such as the red frequency bands in the range of 119 Hz to 614 Hz, and the red region located at 6732 Hz and 184 frames. Thus, Thin ResNet-34 can generate powerful frame-level features for speaker verification.

Figure 5c,d indicate the effectiveness of temporal-frequency aggregation methods in capturing speaker-dependent information. The comparison of the two figures with Figure 5a,b leads to the following observations:

1. In the time domain, the speech regions are assigned higher attention scores, such as the frames from 32 to 56 in Figure 5a, while the non-speech regions are assigned lower attention scores, such as the frames from 216 to 264 in Figure 5a. This indicates that SAP-SGFSAP-19 and ASP-SGFSAP-1 can adaptively emphasize speech frames and suppress non-speech frames.
2. In the frequency domain, there are two red highlighted frequency bands in Figure 5c,d. In terms of speech production, it is believed that the first frequency band in Figure 5c and second frequency band in Figure 5d emphasize the fundamental frequency ranging between 100 Hz to 400 Hz. The fundamental frequency is a speaker-dependent characteristic, and it depends on the length and stiffness of the vocal folds [28]. It should be noted that SAP-SGFSAP-19 and ASP-SGFSAP-1 emphasize different frequency bands in the low-frequency region. This is because the CNN frontend of the two SV systems encodes fundamental frequency information into different bands of frame-level features. Furthermore, the frequency region of 4 kHz–5.5 kHz is associated with the piriform fossa module [28]. This is an important clue for speaker verification, and it is emphasized by the 13th frequency band in Figure 5c,d. In addition, there are some red highlighted spots in Figure 5c,d, which can capture some important formants of vowels, such as /IH/ (average at 1990 Hz) and /EH/ (average at 1840 Hz) in the word “mistake”.
3. Most of the phonetic discriminative information is concentrated in the region from 0.5 kHz to 3.5 kHz [28], which contributes little to speaker verification. Figure 5c,d indicate that the frequency bands in this region are assigned very low attention scores (i.e., the blue region from frequency bands 3 to 11 in Figure 5c,d) to suppress irrelevant information.

Overall, the proposed temporal-frequency aggregation methods can emphasize the speaker-dependent information and suppress irrelevant information in both the time domain and frequency domain. Based on this, they can form a discriminative utterance-level representation to improve the performance of the CNN-based speaker verification systems.

## 6. Conclusions

In this paper, a novel frequency aggregation method called shared-parameter grouped frequency self-attentive pooling (SGFSAP) is proposed for speaker verification. To fully capture the speaker-dependent information contained in the frequency domain, the frame-level features along the temporal axis are grouped first, and a self-attention mechanism is utilized in each group to focus on more informative frequency bands. The shared-parameter method is also adopted to introduce group-invariance into SGFSAP to capture the speaker-dependent frequency information generated by the same phonetic contents in different groups. In addition, by combining SGFSAP with attention-based temporal aggregation, two temporal-frequency aggregation methods are developed to efficiently capture the speaker-dependent information contained in both the time domain and frequency domain of frame-level features. The experimental results on TIMIT and Voxceleb indicate the superior performance of the proposed temporal-frequency aggregation methods to other existing methods. Additionally, the proposed CNN-based SV systems achieve significant improvements compared to the state-of-the-art baselines. In addition, the visualization of attention maps shows that temporal-frequency aggregation methods can emphasize the speaker-dependent information while suppressing the irrelevant information in both the time domain and frequency domain. Future work will focus on the combination of SGFSAP with dictionary-based aggregation methods and the development of powerful CNN architectures to obtain more discriminative frame-level features. Additionally, we will extend temporal-frequency aggregation methods to other speech applications, such as language recognition and emotion recognition.

## Figures and Tables

**Table 1:** The architecture of Thin ResNet-34. ReLu and batch normalization layers are not shown.

Log Fbank Feature ( 1×64×T )	Output Size ( C×H×T )
Conv2d, 7×7 , 16, stride 1	16×64×T
$\left[\begin{array}{c} 3\times 3,16\\ 3\times 3,16 \end{array}\right]\times 3$	16×64×T
$\left[\begin{array}{c} 3\times 3,32\\ 3\times 3,32 \end{array}\right]\times 4$	32×32×TT22
$\left[\begin{array}{c} 3\times 3,64\\ 3\times 3,64 \end{array}\right]\times 6$	64×16×TT44
$\left[\begin{array}{c} 3\times 3,128\\ 3\times 3,128 \end{array}\right]\times 3$	128×16×TT44

**Table 2:** The experimental results on TIMIT under different SNR levels and typical distortions.

Systems	Clean	30 dB	20 dB	10 dB	Distortions
TAP	5.22	5.47	7.01	8.78	8.09
SAP	4.79	5.49	6.84	9.00	8.36
ASP	4.99	5.41	6.69	9.54	8.09
SAP-SGFSAP-10	5.46	5.21	6.50	8.14	7.48
ASP-SGFSAP-2	5.05	5.00	6.62	7.95	9.25

**Table 3:** The experimental results of various SV systems on Voxceleb.

Categories	Systems	EER (%)
GMM-based systems	GMM-UBM [15]	15.0
	i-vector/PLDA [15]	8.8
TDNN-based systems	x-vector (Cosine) [37]	11.3
	x-vector (PLDA) [37]	7.1
CNN-based systems	CNN-embedding [15]	7.8
	SGFSAP-19	6.26
	SAP-SGFSAP-19	6.11
	ASP-SGFSAP-1	5.96

**Table 4:** The experimental results of CNN-based SV systems with different aggregation methods.

Categories	Systems	EER (%)
Temporal aggregation	TAP	6.60
	SAP	6.58
	ASP	6.54
	NetVLAD	7.00
	GhostVLAD	7.14
Frequency aggregation	SGFSAP-19	6.26
Temporal-frequency aggregation	SAP-SGFSAP-19	6.11
	ASP-SGFSAP-1	5.96

**Table 5:** Effectiveness of shared-parameter.

Systems	EER (%)
GFSAP-19	6.40
SGFSAP-19	6.26
SAP-GFSAP-19	6.30
SAP-SGFSAP-19	6.11

## Data Availability

Not applicable.
